# Rickety Curvatures of the Legs

**Published:** 1898-09-10

**Authors:** A. H. Tubby

**Affiliations:** Senior Assistant Surgeon and in charge of the Orthopædic Department, Westminster Hospital, Surgeon to the National Orthopædic and Evelina Hospitals


### Medical Progress and Hospital Clinics.

[*The Editor will be glad to receive offers of co-operation and contributions from members of the profession. All letters should be addressed to* The Editor, at the Office, 28 & 29, Southampton Street, Strand, London, W.C.]

### RICKETY CURVATURES OF THE LEGS.

By A. H. Tubby, M.S.Lond., F.R.C.S.Eng., Senior Assistant Surgeon and in charge of the Orthopædic Department, Westminster Hospital, Surgeon to the National Orthopædic and Evelina Hospitals.

Of all the deformities arising from rickets bow-legs are the most common. To the touch the bones are tender and unusually elastic. The three stages of osseous rickets, namely, congestion, softening, and eburnation, are best seen in the bones of the leg. If one feels the crest of the tibia, the outline is sharp, and the crest itself is deflected. The inner surface of the tibia is concave, and the curvature is rarely purely anterior, being antero-lateral, either externally or internally. The tibia is flattened from side to side, and the curve is generally most marked at the lower third. The medullary canal is often narrowed in the middle of the shaft, and enlarged at the extremities, but occasionally it is dilated throughout its whole extent. When the curvature is very marked, the medullary canal is eccentric, being nearer the convexity than the concavity of the bones. It may be so near the surface of the convex portion as to be separated from it only by a thin layer of bone, or it may actually open on the surface of the bone. On the concave side the bone is much thickened by sub-periosteal deposit, which acts as a supporting buttress to the arch of the concavity. Frequently genu varum and curvature of the femur are present as well, but the chief curve is in the tibia. Curved tibiæ are sometimes associated with genu valgum. The most common co-existing deformity is flat feet.

The age of onset of the deformity is between the first and second year: In rare cases it appears as early as the tenth month, or as late as the third year; but taking the average age of 500 cases brought to me at the hospital, I find it to be two years and two months. In private practice the attention of the medical man is drawn to the deformity in the children of the well-to-do much earlier than in the case of the poorer classes.

The tibia may be curved in almost any direction; but apart from genu varum and curvature of the femur, the following types are found:—

(*a*) An external curvature, generally situated at the junction of the middle and lower third of the leg. With this there is sometimes a twist in the bones, the lower third of the leg looking inwards and forming a well-marked angle externally, with the remainder of the diaphysis. In other cases the curve is chiefly at the junction of the upper and middle thirds of the legs, and the angle is prominent internally. The feet are flat, and the knees are apparently close together. This variety is often mistaken for primary genu-valgum. But if the case be watched from the first, the curvature of the tibia precedes the approximation of the knees.

(*b*) A more or less anterior curvature of the tibia, occupying the whole length of the bone, or only the upper or lower third. The heel is often raised, the foot pointed, and in walking it is in a position of equino-valgus.

(*c*) An internal curvature is present, with flattening of the bones and the feet in a varoid position.

Of these three types the first is common and the third rare. Occasionally one sees an internal curve in one leg, and an external curve in the other.

**Prognosis**: In bow-legs there is always an inclination to spontaneous rectification. This in slight cases is often complete, but in severe cases only partial. It is therefore unwise to allow any case to pass untreated, since if the bones are soft slight cases may very quickly become severe.

**Treatment**: The line of treatment depends upon the condition of the bones, whether soft or eburnated, the direction of the curve, and the age and social status of the patient. In the case of the neglected children of the poor, osteotomy is called for in less severe cases, and earlier than in children of the well-to-do, who obtain efficient supervision and suitable apparatus. All forms of curvature except the marked anterior are amenable to mechanical treatment when the bones are soft. The question of treatment may be discussed under three headings:—

1. Constitutional Treatment with Local Manipulation: This form of treatment is advisable for babies who have not yet walked, as it is a fact that curvature of the tibia and fibula is sometimes present before the child has attempted to walk; for children who are not weighty in the body; for those in whom the bones are not unduly soft, and the curve is a general rather than a localised one.

The usual constitutional means of alleviating rickets must be carried out fully, while the legs should be bathed and rubbed night and morning, and the nurse instructed to hold the leg at the knee and ankle, and, using the thumbs as a fulcrum, to make gentle attempts to straighten the leg. This manipulation should be performed night and morning. A record of the curvature, by means of either photographs or tracings, ought to be taken from time to time, and if the curve show any increase, mechanical supports are required.

2. Constitutional Treatment with Mechanical Supports and Manipulation: This form of treatment is called for when a curve, originally slight, is becoming marked; when a child is weighty, and cannot be kept off his legs; when the curve is located in one part of the bone more than another; and when the child is under four years and the bones are not hardened.

The question arises, should the form of apparatus be such as to entirely prevent the child walking? I think not in any case. All the forms of apparatus act on the principle of taking their fixed points from two bony prominences and drawing the curve towards the support. Provided this is efficiently done, the child should be allowed to use his legs, as free movement by them encourages that improved nutrition which more than counterbalances any of the bad effects of the body-weight.

The simplest form of apparatus is an inside wooden splint from the internal condyle to the internal malleolus for external curvatures, and the reverse for an internal curvature. But in many cases the deflection of the bone is as much anterior as lateral. The single splint is then inefficient, and the difficulty may be overcome by using a trough splint of the following construction: Two straight pieces of wood or tin, of suitable length and width, are joined together so as to make an elongated rectangular splint. If the curve is antero-external, the splint is put on the inside and the back of the leg only. By the pressure of the bandage the antero-external curative is drawn towards the angle of the splint. The same principle exists in the less cumbrous but more expensive tibial instrument, in which there are rigid lateral and posterior rods fixed to the boot and knee-piece, with straps passing round the leg.

In those cases where a marked anterior curvature exists, with elevation of the heel, mechanical appliances are of little or no value.

3. Operative measures: Operative interference is called for when the bones are so hard that mechanical treatment is out of the question—in children over four years of age, in cases of severe anterior curvature, and in marked instances of lateral curvature.

With regard to the choice of operation, the majority of surgeons prefer osteotomy, but some elect to perform osteoclasis. It will be urged that in osteoclasis the fracture is simple, but with correct antiseptics the dangers of sepsis after osteotomy are very remote. Certain it is that osteoclasis is inadmissible (1) if the curvature is very near a joint or epiphysis; (2) if the bone is very hard or firm; (3) if there are several curves in a limb; and (4) if the curve is anterior.

I cannot help thinking that, despite guarded assertions to the contrary, considerable damage may be done to the soft tissues by the use of osteoclasts. In France the Colin's osteoclast is much employed, and in this country Dr. N. Grattan, of Cork, perfected an instrument of extreme power and of great precision.

(*a*) **Manual Osteoclasis.**—Mr. R. W. Murray, of Liverpool, who has had very extended experience of this method, having straightened no less than 311 legs in one year by it, gives the following directions: "If it is a simple curve, to break the bone at its greatest convexity one should not attempt to break it as one would a stick; but grasping the limb, say it is the right leg, firmly at the point at which you wish to produce the fracture, and keeping the left hand perfectly steady, using the thumb as a fulcrum, slowly over-correct the deformity with the right hand until the bone is fractured. It is important that the bone should be fractured and not merely bent, for the tendency of the bent bone to resume its former position is so great that the bandages are apt to become tight and the foot to swell, or the child's delicate skin to blister at the points where the splint presses. If, however, in attempting to straighten bone one has to use so much force as to render it uncertain where the fracture will take place, then, as the object is to straighten the bone and not simply to break it, you had better desist and perform an osteotomy." In no one of his series of cases has Mr. Murray had a mishap.

It appears, then, that manual osteoclasis is a safe procedure, and saves the trouble and time involved in an osteotomy. But with regard to osteoclasts, if the bone requires that great amount of force to fracture it which these instruments are capable of exercising, the writer would prefer osteotomy.

(*b*) **Osteotomy.**—The operation is a simple one. The leg is duly asepticised, and supported on a sandbag. If it be the left leg, the surgeon stands on the left side and passes an osteotomy knife on the flat through the skin over the crest of the tibia, and at the most prominent part of the curve, down over the inner surface of the bone, and then turning it at right angles, firmly grooves the periosteum. Along the knife the saw is introduced, and, as the knife is withdrawn, the skin incision is enlarged to prevent the heel of the saw abrading it. With short movements the tibia is divided, taking care not to wound the structures posterior to it. There is no necessity to divide the fibula with the saw, since, when the section of the tibia is nearly complete, a firm movement inwards or outwards, as the case may be, will fracture the fibula and the remaining portion of the tibia. A counter-opening may be made for drainage. The wound is dressed, and the limb is put up in plaster of paris, and kept so for six weeks.

(*c*) **Removal of a Wedge from the Bone.**—An open wound is necessary in this case. The size of the wedge to be removed should be determined previously by drawing an outline of the leg on paper, and then removing with scissors a sufficient wedge of the paper, so that the model is made straight. In this form of operation the osteotome is desirable, as it is easier to remove the wedge with it than with the saw. Some difficulty may be experienced, after excision of the piece of bone, in getting the leg straight. This arises from two causes—(1) the periosteum on the posterior surface of the tibia is imperfectly divided; (2) in cases of great anterior curvature the tendo Achillis is too short, and prevents apposition of the fragments. It should therefore be divided.

**Non-union after Operations.**—This is so rare an event, considering the enormous number of osteotomies performed, that it is of little practical moment.

